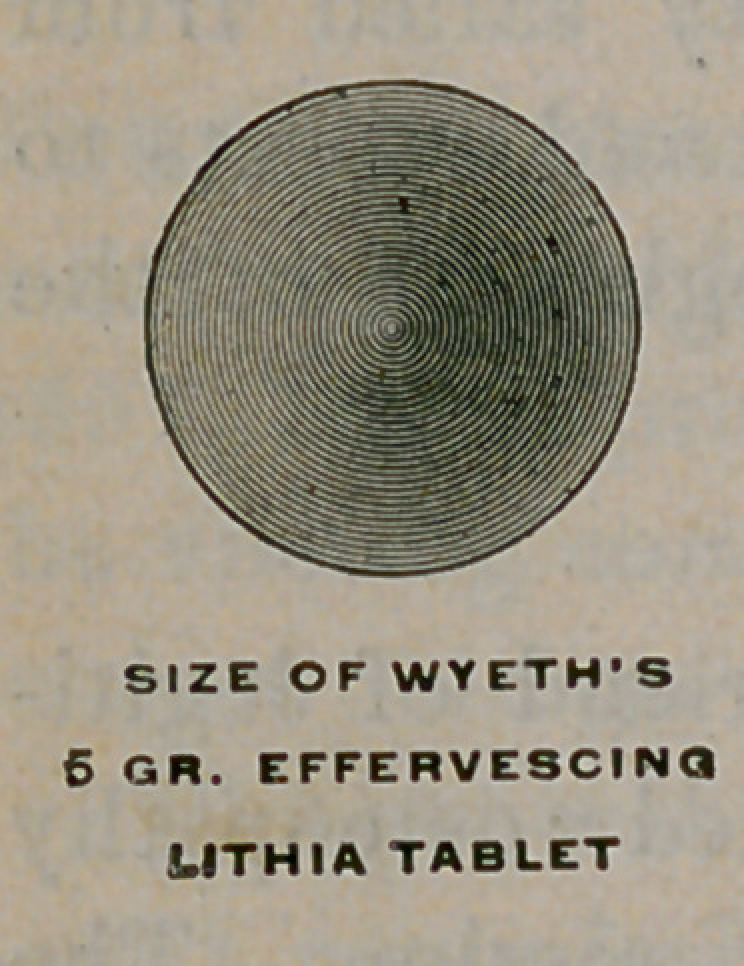# Items

**Published:** 1897-08

**Authors:** 


### Items.

THE ATTACK ON VIVISECTION.—At the meeting of the American Medical Editors' Association, held at Philadelphia, this subject was discussed and a protest was made. against the passage of senate bill 1063, which is entitled For the further prevention of cruelty to animals in the district of Columbia. The bill, under an apparently inoffensive title, endeavors, under the guise of a local measure, to control all experimentations upon animals of whatever nature, and it will, if enacted, prohibit almost absolutely certain lines of experimentation and materially restrict others, both of which we, as scientific men, deem absolutely necessary for the advancement of medicine. The bill is opposed by all of the important scientific bodies in the United States for the reasons : First—Further legislation is unnecessary, the provisions of existing law being sufficient to entirely prevent such cruelty as is mentioned in the bill. The promoters of the bill do not attempt to show that any cruel or unnecessary procedures have been, or are being, performed within the district. Second—The voice of science and medicine is opposed to legislation of any kind which would take in any measure the direction or control of experimental medicine and physiology out of the hands of those who, on account of their special fitness, have been chosen by the authorities of our higher institutions of learning and of research to convey instruction and to conduct investigations at those institutions.

---

An army medical board will be in session at Washington City, D. C., during October, 1897, for the examination of candidates for appointment to the medical corps of the United States army to fill existing vacancies. Persons desiring to present themselves for examination by the board will make application to the secretary of war before September 1, 1897, for the necessary invitation, giving the date and place of birth, the place and state of permanent residence, the fact of American citizenship, the name of the medical college from which they were graduated and a record of service in hospital, if any, from the authorities thereof. The application should be accompanied by certificates based on personal acquaintance from at least two reputable persons as to citizenship, character and habits. The candidate must be between 22 and 29 years of age and a graduate from a regular medical college, as evidence of which his diploma must be submitted to the board. Successful candidates at the coming examination will be given a course of instruction at the next session of the Army Medical School, beginning November 1, 1897. There are five vacancies in the corps to be filled. Further information regarding the examinations may be obtained by addressing Brig.-Gen. George M. Sternberg, M. D., surgeon-general U. S. army, Washington, D. C.

---

The Grand Army of the Republic, in preparing for its encampment to be held in Buffalo during the week beginning August 23, 1897, has to provide for the efficient medical and surgical care of the visiting comrades—not a trifling matter as all previous experience would go to prove.

The general committee at the outset placed Dr. Ernest Wende, health commissioner, at the head of the medical department, and he has appointed 200 physicians to assist in the work. The executive medical staff is as follows: Ernest Wende, medical director; William Warren Potter and Henry Reed Hopkins, assistant medical directors; Albert H. Briggs, director of hospitals; Walter D. Greene, director of sanitation; Geo. W. York, director of ambulances ; Chauncy P. Smith, executive officer and secretary to the committee.

The executive staff is holding weekly meetings (on Wednesdays) at Grand Army headquarters in Ellicott Square and is putting forth plans to give a prompt and efficient medical service to the visiting comrades. Hospitals will be established at appropriate points, with thorough equipment and experienced medical officers and nurses. Ambulances will be at quick command, sanitary inspection will prevent unnecessary sickness, sanitary water-closets will be placed at convenient points, and during the parade the service will be rendered as complete as it is possible for human judgment and precaution to establish.

---

A DELEGATION of Mexican physicians, en route to the Moscow Congress, passed through Buffalo Sunday evening, July 25, 1897. They were accompanied in many instances by members of their families, and the party altogether numbered fifty-three, in charge of Mr. E. O. Matthews, special representative of the Mexican Central Railway. Among them are Dr. E. Lavista, president of the Mexican Medical Association, with his wife and three children ; Dr. Eduardo Licéaga, with his wife and four children ; Drs. Bernaldez, Gavino, Prieto, Garua Diego, Carraza and wife, Domingo Orvanoz and Miss Orvanoz, Jose Morales and Miss Morales, Chavez, Carmona y Valle, Sosa y Gomez, Luis Caimona, Tejeda and wife and two daughters, Vallejo, L. Garibi, F. Garibi and Alonso.

Dr. Licéaga will be remembered by many Buffalonians as the genial president of the American Public Health Association during its meeting in this city last year. This entire party sailed from New York Thursday, July 29th, on the Normannia, and expects to reach Moscow August 13th, five days before the beginning of the Twelfth International Medical Congress.

---

GOVERNOR TANNER, of Illinois, proved himself a friend of legitimate medicine when he affixed his veto to the so-called "osteopathy bill," that passed the Illinois Legislature at its last session. The profession of medicine should everywhere gratefully remember Governor Tanner's action.

---

THE Clinical Society, of Louisville, in conjunction with other physicians of prominence, tendered a banquet, June 22, 1897, to a number of distinguished physicians of that city who had recently been elected or appointed to office in state and national medical organisations. The names of the guests are as follows; Dr. George W. Griffiths, Surgeon-General Kentucky State Militia; Prof. Joseph M. Mathews, M. D., president-elect of the Kentucky State Medical Society and first vice-president elect of the American Medical Association; Prof. William L. Rodman, M. D., chairman of the section on surgery and anatomy in the American Medical Association, and Prof. William Bailey, M. D., vice-president-elect of the National Confederation of State Medical Examining and Licensing Boards. The banquet was presided over by Dr. Bailey, and according to the *American Practitioner and News* the viands and the potations were fitted to the medical gustatory sense, and the "feast of reason and flow of soul" engendered by the occasion was ordered as follows:

Professional fellowship, Dr. William Bailey; The American medical association, Dr. J. M. Mathews, vice-president—response by Dr. L. S. McMurtry; The surgeon-general of Kentucky, Dr. George W. Griffiths—response by Dr. I. N. Bloom; The surgical section, A. M. A., Dr. William L. Rodman, chairman—response by Dr. A. M. Cartledge; The medico-chirurgical society, Dr. J. B. Marvin; The clinical society, Dr. T. P. Satterwhite; The surgical society, Dr. H. H. Grant; Medical journalism, Dr. H. A. Cottell; The Mississippi Valley medical association, Dr. T. H. Stucky.

---

THE Sweetwater Hotel, Hayden Park, Bedford Springs, Mass., was thrown open for guests June 1, 1897. It is a beautiful and substantial house and its proximity to Bedford Mineral Springs makes it a delightful place for summer rest and recreation.

---

SIZE OF WYETH'S 5 GR. EFFERVESCING LITHIA TABLET

EFFERVESCENT lithia tablets afford a convenient form of administering a useful remedy. Instead of bulky bottles of lithia water of variable medicinal virtues, tablets representing a given quantity of lithia, can be dropped into a glass of water when a refreshing as well as a medicinal drink is afforded. Physicians greatly appreciate this and show their favor by adopting in the demands of daily practice the use of these effervescent lithia tablets, giving an expressed preference in prescribing, in many instances, to the products of the Messrs. John Wyeth & Brother, chemists, of Philadelphia, whose art and skill in the science of preparing medicines has gained for them such a highly complimentary reputation.

## Figures and Tables

**Figure f1:**